# Supplementary material for: U-Net Based Segmentation and Characterization of Gliomas
Source: Cancers (Basel). 2022 Sep 14;14(18):4457. doi: 10.3390/cancers14184457 (PMC9496685; doi:10.3390/cancers14184457)
Supplement: Supplementary file 1 [file cancers-14-04457-s001.zip › cancers-1854708-supplementary.pdf]

Table S1. Feature list for model development.

Gender	original_firstorder_TotalEnergy	original_glcm_JointAverage	original_gldm_LowGrayLevelEmphasis	original_glszm_SizeZoneNonUniformity
Age	original_firstorder_Uniformity	original_glcm_JointEnergy	original_gldm_SmallDependenceEmphasis	original_glszm_SizeZoneNonUniformityNormalized
original_shape_SurfaceVolumeRatio	original_firstorder_Variance	original_glcm_JointEntropy	original_gldm_SmallDependenceHighGrayLevelEmphasis	original_glszm_SmallAreaEmphasis
original_shape_VoxelVolume	original_glcm_Autocorrelation	original_glcm_MCC	original_gldm_SmallDependenceLowGrayLevelEmphasis	original_glszm_SmallAreaHighGrayLevelEmphasis
original_firstorder_10Percentile	original_glcm_ClusterProminence	original_glcm_MaximumProbability	original_glrlm_GrayLevelNonUniformity	original_glszm_SmallAreaLowGrayLevelEmphasis
original_firstorder_90Percentile	original_glcm_ClusterShade	original_glcm_SumAverage	original_glrlm_GrayLevelNonUniformityNormalized	original_glszm_ZoneEntropy
original_firstorder_Energy	original_glcm_ClusterTendency	original_glcm_SumEntropy	original_glrlm_GrayLevelVariance	original_glszm_ZonePercentage
original_firstorder_Entropy	original_glcm_Contrast	original_glcm_SumSquares	original_glrlm_HighGrayLevelRunEmphasis	original_glszm_ZoneVariance
original_firstorder_InterquartileRange	original_glcm_Correlation	original_gldm_DependenceEntropy	original_glrlm_LongRunEmphasis	original_ngtdm_Busyness
original_firstorder_Kurtosis	original_glcm_DifferenceAverage	original_gldm_DependenceNonUniformity	original_glrlm_LongRunHighGrayLevelEmphasis	original_ngtdm_Coarseness
original_firstorder_Maximum	original_glcm_DifferenceEntropy	original_gldm_DependenceNonUniformityNormalized	original_glrlm_LongRunLowGrayLevelEmphasis	original_ngtdm_Complexity
original_firstorder_MeanAbsoluteDeviation	original_glcm_DifferenceVariance	original_gldm_DependenceVariance	original_glrlm_LowGrayLevelRunEmphasis	original_ngtdm_Contrast
original_firstorder_Mean	original_glcm_Id	original_gldm_GrayLevelNonUniformity	original_glrlm_RunEntropy	original_ngtdm_Strength
original_firstorder_Median	original_glcm_Idm	original_gldm_GrayLevelVariance	original_glrlm_RunLengthNonUniformity	original_glrlm_ShortRunHighGrayLevelEmphasis
original_firstorder_Minimum	original_glcm_Idmn	original_gldm_HighGrayLevelEmphasis	original_glrlm_RunLengthNonUniformityNormalized	original_glrlm_ShortRunLowGrayLevelEmphasis

original_firstorder_Range	original_glcm_Idn	original_gldm_LargeDependenceEmphasis	original_glrlm_RunPercentage	original_glszm_GrayLevelNonUniformity
original_firstorder_RobustMeanAbsoluteDeviation	original_glcm_Imc1	original_gldm_LargeDependenceHighGrayLevelEmphasis	original_glrlm_RunVariance	original_glszm_GrayLevelNonUniformityNormalized
original_firstorder_RootMeanSquared	original_glcm_Imc2	original_gldm_LargeDependenceLowGrayLevelEmphasis	original_glrlm_ShortRunEmphasis	original_glszm_GrayLevelVariance
original_firstorder_Skewness	original_glcm_InverseVariance	original_glszm_LowGrayLevelZoneEmphasis	original_glszm_LargeAreaHighGrayLevelEmphasis	original_glszm_HighGrayLevelZoneEmphasis
original_glszm_LargeAreaLowGrayLevelEmphasis	original_glszm_LargeAreaEmphasis			

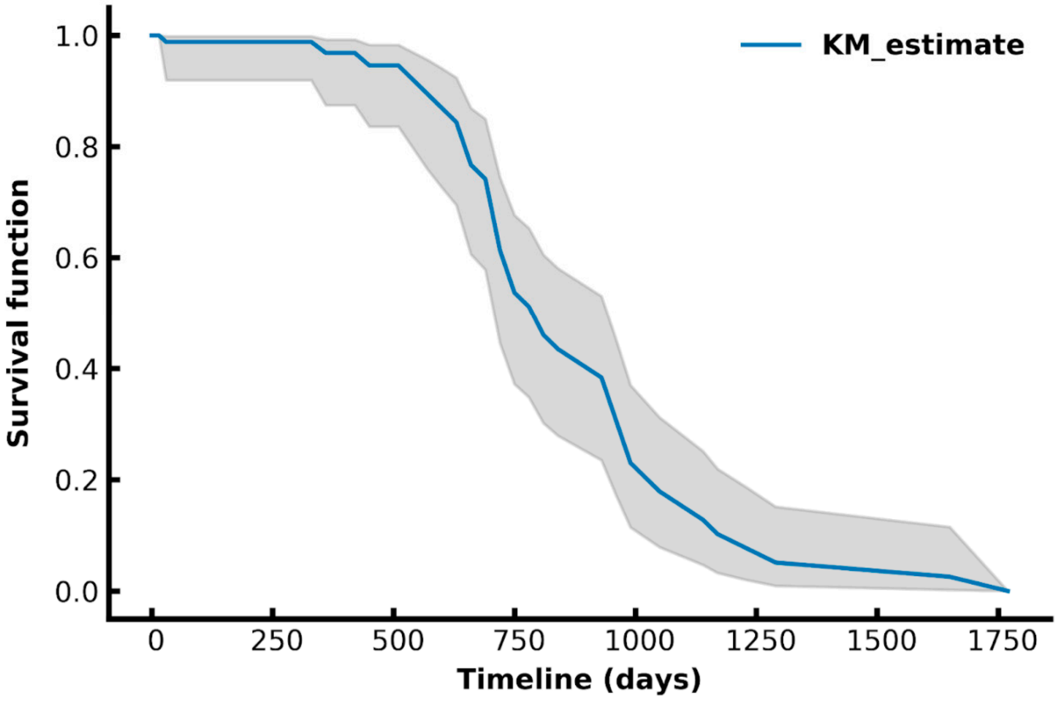

Figure S1. Kaplan Meier curve of survival estimation (MSH n = 89).
